# Potential Drug Targets for Ceramide Metabolism in Cardiovascular Disease

**DOI:** 10.3390/jcdd9120434

**Published:** 2022-12-02

**Authors:** Jiaying Guo, Jiling Feng, Huiyan Qu, Hongxi Xu, Hua Zhou

**Affiliations:** 1 Institute of Cardiovascular Disease of Integrated Traditional Chinese and Western Medicine, Shuguang Hospital Affiliated to Shanghai University of Traditional Chinese Medicine, No. 528, Zhangheng Road, Shanghai 201203, China; 2 School of Pharmacy, Shanghai University of Traditional Chinese Medicine, No. 1200, Cailun Road, Shanghai 201203, China; 3 Engineering Research Center of Shanghai Colleges for TCM New Drug Discovery, No. 1200, Cailun Road, Shanghai 201203, China

**Keywords:** cardiovascular diseases, ceramide, metabolism, drug target

## Abstract

Cardiovascular disease poses a significant threat to the quality of human life. Metabolic abnormalities caused by excessive caloric intake have been shown to lead to the development of cardiovascular diseases. Ceramides are structural molecules found in biological membranes; they are crucial for cell survival and lipid metabolism, as they maintain barrier function and membrane fluidity. Increasing evidence has demonstrated that ceramide has a strong correlation with cardiovascular disease progression. Nevertheless, it remains a challenge to develop sphingolipids as therapeutic targets to improve the prognosis of cardiovascular diseases. In this review, we summarize the three synthesis pathways of ceramide and other intermediates that are important in ceramide metabolism. Furthermore, mechanistic studies and therapeutic strategies, including clinical drugs and bioactive molecules based on these intermediates, are discussed.

## 1. Introduction

Cardiovascular diseases (CVDs) rank first in global morbidity and mortality, accounting for 40% of deaths according to a prospective cohort study of common diseases, deaths, and hospitalization rates among middle-aged (35–70 years) individuals in 21 countries in 5 continents [1,2]. In recent years, the incidence of metabolic abnormalities has continued to increase, owing to excessive caloric intake and environmental pollution [3], and contributes to the incidence and severity of a range of diseases, including coronary atherosclerosis, cardiomyopathy, heart failure, type 2 diabetes, and hypertension [4,5].

Metabolic flexibility is among the physiological characteristics of a healthy heart. It allows the heart to arbitrarily use various energy sources to better adapt to different environmental stresses. The main energy source of the heart is long-chain fatty acids (FAs). However, in a failing heart, the β-oxidative capacity of FAs is inhibited, which makes glucose the main energy source, leading to further accumulation of FA intermediate metabolites [6]. When FA accumulates excessively in the circulation, it leads to impaired oxidative pathways, further leading to the accumulation of undesirable metabolites, including ceramide [7]. In turn, a portion of the deleterious ceramide is subsequently overproduced and induces apoptosis in cardiomyocytes, closely associated with left ventricular systolic dysfunction and impaired filling, which is common in hyperlipidemic and hyperemic cardiomyopathies, and diabetic states [8]. Ceramide has been considered as a predictor of CVD, and may be a more accurate indicator than low-density lipoprotein (LDL) cholesterol [9,10,11].

It is important to note that sphingolipids are a complex and diverse group of lipids. Because of their structural and metabolic diversity, not all sphingolipids are detrimental. Studies have shown that glycosphingolipids, in addition to playing an important role in maintaining the structure of cardiac myocytes, are necessary for their function as well as for the long-term maintenance of normal cardiac function [12]. Therefore, they should be taken into account in scientific research. This paper focuses on the cardiovascular effects of harmful ceramides and their metabolic pathways, as well as reviews the current studies in this field.

Ceramides present in circulating LDL have been shown to cause and exacerbate insulin resistance [13]. One mechanism by which ceramide production leads to cellular damage is that it disrupts the activity of the electron transport chain and affects mitochondrial respiration, leading to impaired energy metabolism [14]. As ceramide accumulates in the outer mitochondrial membrane, it increases the permeability of cytochrome C, and eventually triggers apoptosis [15]. At the same time, ceramide can also lead to impaired cellular function by affecting the balance of cellular autophagy. Ceramide produced by metabolic disorders activates protein kinase B (Akt, or PKB) and pro-apoptotic signals, which in turn leads to impaired AKT-stimulated glucose transporter protein ectopic in the cell membrane, thereby inhibiting glucose uptake and glycogen synthesis, and leading to the development and progression of insulin resistance and type 2 diabetes [16].

In addition to acting directly on cardiac muscle cells, ceramide acts on other cells to further affect cardiac function. Vascular endothelial cells are dysfunctional in both heart failure and coronary artery disease, and researchers have found that ceramide acts directly on endothelial cells (ECs), preventing vasodilation and exacerbating the risk of cardiovascular disease [17]. In animal and cellular experiments, administration of ceramide analogs impaired endothelium-dependent vasodilation [18], reduced nitric oxide production [19], exacerbated vasoconstriction [20], and harmed cardiovascular function. In this article, we review the progress of laboratory and clinical research on ceramide modification related to the prevention and treatment of CVD.

## 2. Ceramide Biosynthesis and Metabolism

**Figure 1:**
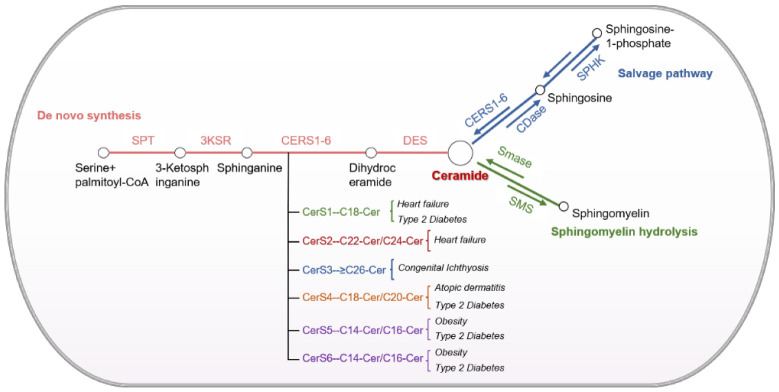
Major ceramide synthesis and metabolic pathways in cardiovascular system: De novo synthesis pathway, sphingomyelin hydrolysis pathway, and the salvage pathway. The rate-limiting enzyme serine palmitoyltransferase (SPT) condenses palmitoyl coenzyme A and serine to form 3-ketosphinganine, then is rapidly reduced to sphingosine by 3-ketosphinganine reductase (3KSR). Sphingosine is acetylated by ceramide synthase (CERS) to yield dihydroceramide, which is further modified by dihydroceramide desaturase (DES) to produce ceramide. Each CerS uses a relatively limited subsection of fatty acyl-CoAs to acylate sphingoid [22,23]; CerS1 utilizes mainly C18-CoA and is mainly associated with heart failure and type 2 diabetes, while CerS2 has acyl-CoA specificity and utilizes longer acyl-CoA chains (C20-C26) for ceramide synthesis, which is closely associated mainly with heart failure [24,25,26]. CerS3 uses very long acyl-CoA (greater than C26), which affects congenital ichthyosis onset and progression, while CerS4 uses C18- and C20-CoA closely associated with the diseases atopic dermatitis and type 2 diabetes [27,28,29]. In addition, CerS5 and CerS6 are more likely to produce short-chain ceramides, such as C14- and C16-CoA, mainly in obesity and type 2 diabetes [30,31,32,33]. Sphingomyelinase (SMase) hydrolyses sphingomyelin (SM) to ceramide, and SM synthase converts ceramide to SM. Ceramides can also be transformed into sphingosine by the action of ceramidase, and are subsequently phosphorylated by sphingosine kinase (Sphk) to produce the bioactive lipid sphingosine 1-phosphate (S1P). SPT, serine palmitoyltransferase; 3KSR, 3-ketosphinganine reductase; CERS, ceramide synthase; DES, dihydroceramide desaturase; SMase, sphingomyelinase; SMS, sphingomyelin synthase; CDase, ceramidase; Sphk, sphingosine kinase; S1P, sphingosine 1-phosphate.

Sphingolipids, such as ceramide, sphingosine 1-phosphate, and sphingomyelin (SM), are structural molecules found in biological membranes. They maintain the barrier function and fluidity of the membrane, which is important for cell survival, lipid metabolism, and adaptation to cellular stress. Ceramides are an important subgroup of sphingolipids, playing biologically active roles. Furthermore, they are established as second messengers that regulate many key cellular processes [21]. Depending on their chain length, ceramides can be classified as short-chain (C2 to C12-ceramide, indicating an acyl chain of 26 carbon atoms), long-chain (C14 to C18-ceramide), and ultra-long-chain ceramides (C22 to C26-ceramide) [22]. Ceramides are synthesized by enzymes involved in different subcellular compartments, whose functional sites are selectively activated after stimulation by different stresses, leading to the production of ceramide through different pathways [21]. The main pathways of ceramide synthesis are de novo synthesis, sphingomyelin synthesis, and the salvage pathways (Figure 1).

### 2.1. De novo Synthesis

Despite the considerable diversity of the sphingolipid library, the synthesis of these molecules relies on a simple and conservative de novo synthesis pathway consisting of four steps [34]. The ceramide de novo synthesis takes place at the endoplasmic reticulum (ER), and is catalyzed by serine palmitoyltransferase (SPT), which combines palmitoyl coenzyme A and serine to produce 3-ketosphingosine [35]. It is then reverted to dihydrosphingosine and acylated by ceramide synthase (CerS) to dihydroceramide. Ceramide synthases (CerS1-6) are important enzymes that localize in the endoplasmic reticulum (ER), where they aid ceramide synthesis and release the ceramides into the cytosol [36]. On the other hand, CerS acylates sphingosine to produce dihydroceramide [37]. On the ER membrane, dihydroceramide desaturase (DES) further modifies dihydroceramide by introducing an important trans-4,5-enol double bond, ultimately leading to the formation of the corresponding ceramide isoforms [38,39]. After transfer to the Golgi apparatus via the ceramide transporter or vesicular transport, ceramides can be further modified by the addition of hexose, choline, or phosphate groups to produce complex sphingolipids, such as glucosylceramide, sphingomyelin, or 1-phosphoceramide [40].

### 2.2. Sphingomyelin Pathway

The sphingomyelin (SM) synthesis pathway is the second pathway of ceramide synthesis. Upon entering the Golgi apparatus, SM synthase (SMS) converts ceramide to SM. SM hydrolysis causes a rapid and transient increase in ceramide content. On the contrary, the de novo synthesis pathway requires multiple enzymatic steps, and is responsible for the slow but substantial accumulation of ceramides within a few hours [41].

At least two pathways are relevant to the transport of ceramides from the ER to the Golgi apparatus in SM synthesis; a major adenosine triphosphate and cytosol-dependent pathway and a minor independent pathway [42]. SM is generated from ceramides in the presence of SMSs. Two isoforms of SMSs, namely, SMS 1 and 2, have been identified in humans. SMS 1 is located in the trans-Golgi region, while SMS 2 is located on the plasma membrane, possibly promoting the formation of sphingomyelin from ceramides produced at the cell surface level [43]. The phosphodiester bond in SM can be hydrolyzed by sphingomyelinase (SMase) to produce ceramide and phosphorylcholine [44]. SM synthesizes ceramide via SMase activation. SMases are classified according to their optimal pH and subcellular localization. Hence, they are divided into acidic, neutral, and basic subtypes [45,46]. Acidic SMase and neutral SMase play a major role in stress-induced ceramide production in response to apoptosis and stress stimuli [35].

### 2.3. Salvage Pathway

The third metabolic pathway of ceramide synthesis is the salvage pathway. This pathway is mostly activated when the cells are stimulated by environmental stress. Five different categories of ceramidases (CDases), namely, acid, neutral, and alkaline CDase 1, 2, and 3, have been identified based on their optimal pH, as well as their primary structure and function. These CDases are encoded by *asah1* (N-acylsphingosine amidohydrolase 1), *asah2*, *acer1* (alkaline ceramidase 1), *acer2*, and *acer3*, respectively [47]. Alkaline ceramidases are located on cell membranes and Golgi complexes, regulating cellular functions such as cell differentiation and proliferation [48,49]. Neutral CDases mainly function in the cell membrane to modulate intestinal levels of sphingolipid metabolites [50]. Acid ceramidases have received increasing attention in the field of CVD, and are localized to the lysosome, where they maintain intra-lysosomal ceramide homeostasis [51]. ASAH1 deacetylates ceramide to release long-chain sphingosine, which is further phosphorylated to sphingosine 1-phosphate (S1P) by sphingosine kinase 1 or 2 (Sphk1/2). Sphingosine can be reused by ceramide synthase to produce ceramide again [52].

The three metabolic pathways of ceramide interact to allow the interconversion of different sphingolipids, so that all sphingolipids remain in a constant flow between the different isoforms. Among the many sphingolipids, ceramide is considered the hub since it is located at the center of the sphingolipid metabolic pathway. As an important indicator of cardiometabolic diseases, ceramides have emerged as potential targets for improving cardiovascular events.

## 3. Role of Ceramide in Cardiovascular Health

Evidence indicates that ceramides correlate with CVD [53]. Clinical studies have shown that plasma ceramides levels are elevated in patients with heart failure and ischemic cardiomyopathy [54]. A clinical study in Japan showed a positive correlation between the levels of ceramides and lipid biomarkers (total cholesterol, TG, and phospholipids) [55]. An excess supply of FAs in cardiomyocytes (CMs) is correlated with increased ceramide content and increased risk of lipotoxic cardiomyopathy. Furthermore, high ceramide concentrations are associated with myocardial infarction recurrence and mortality [56].

Ceramide levels may also show additional predictive potential. Increased plasma ceramide levels are associated with the severity of chronic heart failure (CHF), while an increase in cardiac remodeling and cardiac dysfunction is correlated with plasma ceramide species [57]. Therefore, plasma ceramide may be a promising diagnostic marker for identifying patients at risk for adverse CVD [56]. Research shows that enrichment of several long-chain and very-long-chain ceramides (C16, C18, C20:1, C20, C22:1, C24:1) in the serum has been identified to correlate with heart failure [58]. Clinical studies, including FINRISK [59], Corogene [9], and PREDIMED [11], indicate that Cer 16:0, 18:0, and 24:1 present a substantially higher risk of CVD. Inhibition of ceramide production has been shown to rescue many other complications caused by obesity and insulin resistance, including type 2 diabetes [60,61], alcoholic [62] and nonalcoholic [63] fatty liver disease, atherosclerosis [64,65], hypertension [66], and cardiomyopathy [54,67]. Moreover, elevated cardiac ceramide levels correlate with ischemic CHF and acute ischemic injury in mice [54]. Cardiolipotoxicity is observed in hyperlipidemia and diabetes caused by lipid metabolism disorders, such as those characterized by the accumulation of ceramides [7,68]. In the rat left anterior descending artery (LAD) model, abdominal aortic blood collected at different time points after myocardial infarction showed increased total ceramide levels in the plasma [69]. Zucker diabetic fatty (ZDF) model rats developed myocardial dysfunction when 14 weeks old. Troglitazone treatment decreased myocardial TG and ceramide levels and reserved cardiac function in ZDF rats [8].

Notably, ceramides are richly classified, and not all types of ceramides are harmful. Using human myocardial biopsies, researchers found elevated levels of glycosylceramide and lactic acid glyceramide during ischemic cardiac remodeling compared to levels in non-ischemic hearts. To clarify the role of glucosylceramide in ischemic myocardium, researchers thus generated mice with cardiomyocyte-specific deficiency in Ugcg, the gene encoding glucosylceramide synthase (hUgcg^−/−^ mice). The findings suggest that critical levels of glycosphingolipids are necessary for the maintenance of β1-adrenoceptor signaling via the endolysosomal system, as well as for the maintenance of cardiomyocyte structure and function, and the long-term maintenance of normal cardiac function [12].

## 4. Mechanistic Studies Based on the Key Steps of Ceramide Synthesis

**Figure 2:**
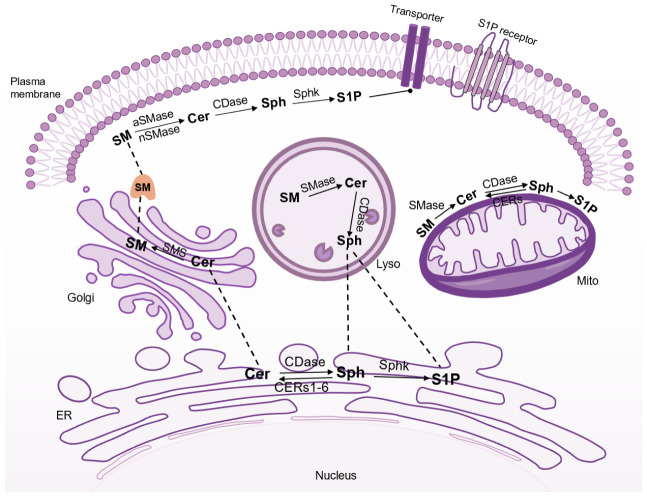
Intermediates involved in the three synthesis processes of ceramide that have the potential to be developed into drug targets. The ceramide de novo synthesis pathway occurs in the endoplasmic reticulum (ER), and is subsequently transported via the ceramide transport protein (CERT) or vesicles to the Golgi apparatus, where ceramide can be metabolized to other types of sphingolipids. The sphingolipid pathway occurs in the Golgi apparatus, mitochondria, and plasma membrane, where sphingosine and ceramide can be interconverted by enzymes. The remedial pathway takes place in lysosomes, where sphingosine 1-phosphate is produced via ceramidase and Sphk.

Several studies have shown that interfering and blocking ceramide synthesis can be an effective strategy for the treatment of cardiovascular diseases [4,5]. A variety of enzymes, intermediates, and proteins play important roles during the three processes of ceramide synthesis (Figure 2). Inhibitors of key enzymes and intermediates, or the knockout of key proteins of the synthesis pathway, can effectively block ceramide synthesis and reduce the content of ceramides.

### 4.1. Key Enzymes and Mediators in the de novo Synthesis Pathway Related to Cardiovascular Diseases

#### 4.1.1. Serine Palmitoyltransferase

The initiation of sphingolipid de novo synthesis requires the rate-limiting enzyme serine palmitoyltransferase (SPT). SPT consists of three genetic isoforms: SPTLC1, SPTLC2, and SPTLC3. SPT is found to be upregulated in damaged myocardia [54].

All isoforms of SPT have been shown to affect a cardiovascular mouse model using genetic methods. SPTLC1 haploinsufficiency blocks sphingolipid biosynthesis and ameliorates the dilated phenotype and contractile function caused by dyslipidemia in cardiomyopathic mice [67]. When exposed to a diabetes-like environment or transfected with SPTLC1, intracellular ceramide levels increase in cardiomyocytes, resulting in increased autophagy and mitochondrial dysfunction [70]. Deletion of SPTLC2 preserves cardiac function after myocardial infarction [54,71]. Hippocampal respiration assay illustrates that the inhibition of SPTLC2 expression can limit the rate of ceramide synthesis and affect ceramide production [54]. However, it has also been shown that SPTLC2 haploinsufficiency leads to a 35% decrease in ceramide and a worsening of cardiac function [72]. SPTLC3 expression is elevated in the myocardium and cardiomyocytes [73], and correlates with plasma ceramide concentrations (C22:0 and C24:0) [74]. In the human cardiomyocyte cell line AC16, SPTLC2 overexpression causes a significant accumulation of C14, C16:1, C16, and C24:1 ceramides. In addition to genetic approaches, pharmacological treatments can also inhibit SPT expression and thus reduce ceramide production. As a high-affinity inhibitor of SPT, myriocin reduces fibrosis and ventricular remodeling after myocardial infarction, decreasing the total ceramide content, which is beneficial for improving insulin sensitivity and attenuating dyslipidemia [54,71].

Increased ceramide levels in acute and chronic ischemic injury are caused by an activated de novo pathway rather than by sphingomyelinase or salvage pathways [54]. Accordingly, modulating the de novo ceramide synthesis pathway may present a novel target for the treatment of metabolic cardiomyopathies [70]. Furthermore, it has been confirmed that the pro-apoptotic effect of ceramide after myocardial infarction can be reduced by inhibiting the de novo synthesis pathway [75]. Intracardiac pre-administration of myriocin selectively inhibits ceramide synthesis and reduces the infarct size by 40.9% [71]. In a LAD coronary ligation mouse model, myriocin treatment reduced the size and mass of the ventricle. After 8 weeks, reduced ceramide secretion alleviates cardiomyocyte-macrophage infiltration and cardiac fibrosis [54].

#### 4.1.2. Ceramide Synthases

Ceramide synthases (CerSs) are core enzymes in the de novo synthesis of ceramides and other sphingolipids. CerS2 overexpression elevates ceramide levels and decreases mitochondrial homeostasis and ATP production [76]. CerS5 can selectively generate C14 ceramide, which accumulates in the heart. CerS5 knockdown has been shown to counteract palmitate lipotoxicity and apoptosis [77], or prohibit myristic-acid-induced cardiomyocyte hypertrophy [78].

#### 4.1.3. Dihydroceramide Desaturase

Dihydroceramide desaturase (DES or DEGS) catalyzes the final step of the de novo synthesis pathway. Inhibition of DES1 enzymatic activity alters ceramide synthesis [79,80]. The use of pharmacotherapy or loss-of-function experiments for genetic ablation of DES1 has been shown to prevent adipogenesis and reduce lipid accumulation. Pharmaceutical inhibition of DES1 has been validated in vivo to impair adipocyte differentiation, providing a potential target for the treatment of lipid metabolism disorders [81].

Des1^−/−^ mice have highly elevated levels of dihydroceramide, and fail to form essential ceramides, which leads to infertility and multiple organ dysfunction [60]. However, it has also been confirmed that phenotypic abnormalities in Des1^−/−^ mice include scaly skin and sparse hair, tremors, and abnormal liver function test results [60]. Prolonged DES1 mRNA deficiency impedes ceramide synthesis and leads to DHC-16-ceramide accumulation, suggesting that DES1 may prove to be a potential target to alleviate ceramide levels under hypoxic stress [82].

### 4.2. Key Enzymes and Mediators in the Sphingomyelin Pathway Related to Cardiovascular Disease

#### 4.2.1. Sphingomyelin

Sphingomyelin (SM) is mainly located in the plasma and organelle membranes. SM can be catalyzed by acidic and neutral sphingomyelinase (SMase) isomers to generate ceramides. Ceramide can be regenerated by SMS. Most mammalian cells use the sphingomyelin pathway as a downstream effector of environmental stressors [83]. The development and progression of CVD is closely related to LDLs, and excessive LDL deposition under the vascular intima is the initial stage of atherosclerosis. SM stored in LDLs can be hydrolyzed to ceramide by SMase, which further promotes the formation of ceramides in the serum and accelerates CVD progression [84]. Thus, improving sphingomyelin metabolism could be a therapeutic approach to treat atherosclerosis.

#### 4.2.2. Sphingomyelin Synthases

Sphingomyelin is produced by SMSs during the transfer of phosphorylcholine. Plasma sphingolipids have been shown to be an independent risk factor for CAD. Therefore, SMS2 knockout mice show reduced plasma SM levels and atherosclerosis in the aortic arch and thoracoabdominal aorta after 3 months of a high-fat diet. In addition, SMS2 knockout mice show reduced nuclear factor kappa B (NF-κB) expression in macrophages, suggesting that SMS2 deficiency reduces atherosclerosis and suppresses inflammation [85]. On the other hand, SMS2 overexpression results in higher sensitivity to hypoxia-induced damage. Dietary Mg^2+^ content is also reported to affect sphingolipid metabolism. Dietary Mg^2+^ deficiency for a short period causes an upregulation of SMS and p53 expression in the cardiac and aortic smooth muscles [86].

#### 4.2.3. Sphingomyelinases

Sphingomyelin can produce ceramides via SMase catalysis. To date, at least five types of SMases have been identified, including acidic SMase (A-SMase), which is ubiquitous in lysosomes and serum, and is Zn^2+−^dependent; neutral SMase (N-SMase), which may be Mg^2+−^dependent; and alkaline SMase [87].

Activation of A-SMase is a major pathway for promoting apoptosis through a multitude of pro-apoptotic signals in different cell types [88]. The researchers obtained the right atrial auricle from 20 patients who underwent coronary artery bypass graft (CABG) surgery, and subsequently simulated in vitro arrest and conducted reperfusion experiments. It was found that ceramide secretion increased after cardiomyocyte damage, and that amitriptyline reduced apoptosis after cardiac arrest and reperfusion by inhibiting acid sphingomyelin [89]. Furthermore, N-SMase activation occurs in infarcted myocardia. In a rat LAD model, ceramide accumulation in the ischemic myocardium inhibited SMase activity and reduced its production after ischemic preconditioning [90].

### 4.3. Key Enzymes and Mediators in the Salvage Pathway Related to Cardiovascular Diseases

#### 4.3.1. Sphingosine 1-Phosphate

Sphingosine 1-phosphate (S1P) is a bioactive lysophospholipid located on the cell membrane [91]. As an important intracellular phospholipid mediator, S1P functions intracellularly as a second messenger, and also extracellularly as a G protein-coupled receptor (GPCR) ligand [92,93]. S1Plyase (S1PL) is an endogenous enzyme that degrades S1P. S1PL inhibition has cardioprotective effects via a reduction in S1P degradation [94]. S1P shares five different receptors (S1PR), of which S1PR 1–3 are ubiquitously expressed. On the other hand, S1PR 4 and 5 are mainly expressed in the immune and nervous systems [95]. Antagonizing the main receptor isoform of S1P in cardiomyocytes, S1PR1, has been shown to protect cardiomyocytes [62].

In addition to protecting the myocardium and enhancing resistance to stress such as ischemia and hypoxia, S1P also contributes to the inhibition of myocardial hypertrophy. For example, S1P pretreatment for 2 min protects against ischemia and reperfusion injury by improving left ventricular systolic and diastolic function and inhibiting the release of creatine kinase [96]. Exogenous S1P has been reported to increase the viability of cardiomyocytes cultured under hypoxic conditions and reduce perfusion in isolated hearts of rats after ischemia/reperfusion [96]. Furthermore, S1P reduces cardiomyocyte hypertrophy and the expression of hypertrophic genes [97].

Unlike sphingomyelin, most (about 85%) lipoprotein-associated S1P in the blood binds to high-density lipoprotein (HDL) [98]. Research has shown that S1P promotes vasodilation, angiogenesis, and endothelial barrier function, thereby protecting against ischemia/reperfusion injury and inhibiting the development of atherosclerosis [99]. Moreover, S1P maintains vascular endothelial barrier function and blood pressure [100]. It is found that after S1P binds to protein G, the complex activates phosphoinositide 3-kinase (PI3K) to activate the pro-survival Akt kinase [62]. S1P directly activates PI3K-Akt and extracellular signal-regulated kinase 1/2 (Erk1/2) signaling [101,102], increases the phosphorylation of Akt and GSK3b, and reduces the opening of the mitochondrial permeability transition pore (MPTP); this opening leads to mitochondrial swelling, loss of mitochondrial membrane potential, and release of cytochrome c from mitochondria to the cytoplasm, resulting in irreversible cell damage [103]. S1P was also reported to activate signal transducer and activator of transcription 3 (STAT3), mainly through the S1P2 receptor, confirming that S1P exerts cardioprotection by activating AKT and STAT3 [104]. Therefore, exploring ways to increase the synthesis of S1P, or reduce its degradation, represents a therapeutic approach for myocardial protection after myocardial injury.

#### 4.3.2. Ceramidases

Acid ceramidase (ACDase), as the enzyme that hydrolyzes pro-apoptotic ceramides to produce sphingosine, has received the most attention among the three ceramidase enzymes [52]. The overexpression of ACDase counteracts the negative effects of elevated ceramide levels, and has been shown to exhibit a cardioprotective effect. ACDase synthetic modified RNA (modRNA) treatment in mice protected cardiac function after myocardial infarction [105]. ACDase modRNA delivery decreased ceramide levels and protected immune cells, indicating that ACDase overexpression aids the transient alteration of sphingolipid metabolism. It has been reported that increased ACDase activity simultaneously decreases ceramide levels and increases S1P levels in diabetic mice and cultured human cardiomyocytes [106].

#### 4.3.3. Sphingosine Kinase

Sphingosine kinases 1 and 2 (SphK1 and 2) catalyze the phosphorylation of lipid sphingosine and generate S1P. SphK/S1P exists in blood vessels and cardiomyocytes, making SphK-related sphingolipid metabolism more direct and immediate [107]. Activation of protein kinase C epsilon (PKCε) and mitochondrial K+−ATP signaling protects cardiomyocytes from ischemia/reperfusion injury [108,109]. A high endogenous SphK1 level has been shown to impede ceramide synthesis, while cells lacking SphK1 accumulated ceramide in the mitochondria, causing mitochondrial events that lead to cell death [110].

In cardiomyoblasts, SphK1 inhibition by pharmacological inhibitors or small interfering RNA enhances oxidative stress caused by C(2)-ceramide or H_2_O_2_ addition, whereas SphK1 overexpression reverses these injuries. It has been reported that the upregulation of the ceramide/S1P ratio is crucial for mitochondria-mediated apoptosis [111]. SphK1-knockout mice in a potassium-induced cardiac arrest resuscitation model show reduced S1P levels in tissues and circulation, leading to poor resuscitation and a reduced post-resuscitation survival rate after cardiac arrest [112]. SphK2 is required for autophagosome and lysosome-mediated intracellular lipid droplet breakdown to impede the development of atherosclerosis. In macrophages, SphK2 deficiency leads to an increase in intracellular sphingomyelin and ceramide levels. The number of autophagosomes and autolysosomes containing lipid droplets in SphK2-deficient macrophages increased, and there was a defect in the degradation of lipid droplets by these lysosomes, resulting in increased sphingomyelin and ceramide in the cells. These results suggest that SphK2 is required for autophagosome- and lysosome-mediated intracellular lipid droplet catabolism to impede the development of atherosclerosis [113]. Therefore, SphK2 may be a novel target for the treatment of cardiovascular disease.

## 5. Sphingolipid Metabolism and Cardiovascular Disease Therapies

Although ceramide plays an important role in CVD, there is currently no clinical treatment targeting ceramide. Clinical lipid-lowering investigations have shown that modifying lipid metabolism can also decrease ceramide concentration. Ceramides are mainly carried by LDLs in the circulatory system, and clinical studies have confirmed that lowering lipid levels using statins can reduce serum ceramide levels [114,115]. With the continuous in-depth research on ceramides, the clinical value of ceramide in clinical diagnosis and treatment has gradually been discovered.

**Figure 3:**
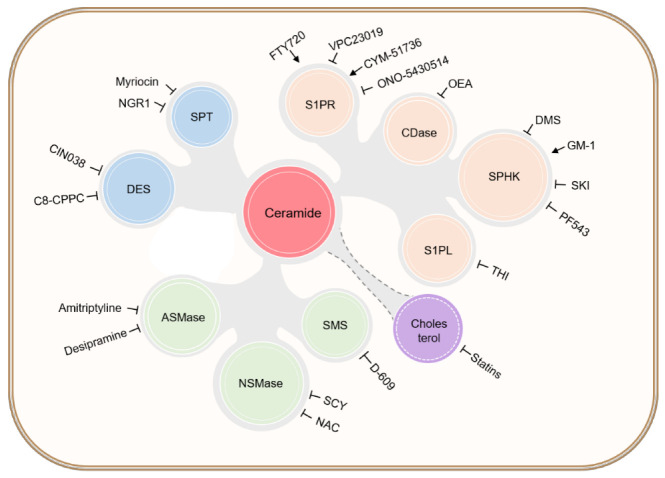
Clinical drugs and bioactive molecules affecting sphingolipid metabolism and their targets. The above drugs can affect sphingolipid metabolism by acting on key enzymes or receptors in the three metabolic pathways. S1PR, S1P receptor; S1PL, S1P lyase; NGR1, notoginsenoside R1; CIN038, 4- ((5-(4-(trifluoromethyl)phenyl)-1,3,4-oxadiazol-2-yl)amino)phenol; C8-CPPC, C8-cyclopropenylceramide; NAC, N-acetylcysteine; SKI, sphingosine kinase inhibitor; GM-1, monoganglioside; DMS, N,N-dimethylsphingosine; OEA, N-oleoylethanolamine; CYM-51736, N,N-dicyclohexyl-5-(furan-3-yl)isoxazole-3-carboxamide; THI, 2-acetyl-5-tetrahydroxybutyl imidazole.

As mentioned above, ceramide has already been used as a biomarker of CVD in large human cohorts. Therefore, it is considered an indicator of disease risk [10]. A Finnish cohort study showed that three ceramide subtypes (C16:0, C18:0, and C24:1) are positively correlated with CHF progress, while C24:0 is negatively expressed [9]. Many other clinical studies also allude to the relationship between ceramide and disease in myocardial tissue. Below, we summarize the research findings on the mechanism of clinical drugs and natural products that have been investigated to regulate ceramide metabolism (Figure 3).

### 5.1. Clinical Drugs Regulating Ceramide Content

Researchers have found that currently available clinical agents have a clear target effect on reducing ceramide levels, which provides ideas for expanding the scope of their use. Fingolimod (FTY720) is a synthetic derivative of myriocin, which is the first drug approved by the United States Food and Drug Administration (FDA) in 2010 for the treatment of multiple sclerosis [116,117]. Thus far, fingolimod is the only drug targeting ceramide synthesis. It is an S1PR1 activator that exerts a cardioprotective effect as an analog of sphingosine. In an ischemia/reperfusion model, fingolimod treatment activated cardioprotective reperfusion injury salvage kinase signaling (RISK) and survivor activating factor enhancement (SAFE) pathways in the infarct border zone of pigs [75]. After prolonged use, fingolimod improved myocardial salvage capacity, reduced infarct size, and enhanced systolic left ventricular function post myocardial infarction [116,117]. Although fingolimod has been generally well tolerated in phase III trials, some patients suffered from cardiac events, including bradycardia and atrioventricular blocks [118,119]. Therefore, the use of ceramide-targeting drugs in the treatment of CVD remains a challenge.

Several other FDA approved drugs are also reported to inhibit ceramide levels, which may contribute to expand their applications [120]. The antidepressant, amitriptyline, is an acid SMase inhibitor that inhibits apoptosis to suppress ischemia/reperfusion injury [89]. Amitriptyline, as an approved tricyclic antidepressant, has completed phase IV trials for genetic pain treatment, or the treatment of other types of pain. Amitriptyline at its therapeutic concentration is also reported to have reduced ceramide concentrations in the hippocampus, which in turn increased neuronal proliferation, maturation, and survival, as well as improved the behavior, of mice in a depression model [121]. Desipramine is an FDA-approved antidepressant that blocks the recovery of monoamine neurotransmitters to treat depression. Administration of desipramine could also partially protect reperfusion [122], indicating that reducing ceramide accumulation in ischemic hearts by inhibiting acid sphingomyelinase has a cardioprotective effect. Desipramine treatment has been shown to eliminate increased ceramide levels, oxidative stress, and elevated troponin I, ultimately improving cardiac insufficiency [123]. Empagliflozin is a routinely used drug for type 2 diabetes. Metabolomics of the myocardial tissue of diabetic fatty rats confirmed that empagliflozin substantially reduces the sphingolipid and glycerophospholipid content of cardiac tissue. Furthermore, empagliflozin modulates the abundance of amino acids associated with tricarboxylic acid supply and cardiac function, confirming its effect in improving myocardial metabolism, inhibiting myocardial inflammation, and reducing apoptosis [124]. Although it has completed phase IV trials to treat diabetes patients, empagliflozin has already opened up phase IV trials for acute heart failure and atrial fibrillation. Recently, a cohort study showed that treatment with SGLT-2 inhibitor, empagliflozin, has lower risk and mortality for heart failure compared with the first-line metformin, suggesting the heart protection function of empagliflozin. Whether inhibiting ceramide can be one of the underlying mechanism of empagliflozin to treat heart disease needs to be clarified [125,126].

Other approved drugs or additives have been shown to similarly modulate ceramide levels, but the targets of action have not been elucidated and need further study. Clinically used lipid-lowering agents, such as statins, are effective in reducing circulating lipid levels, including ceramide levels. A randomized, parallel, three-arm study in subjects treated with the clinically indicated lipid-lowering agents simvastatin, ezetimibe, or their combination, showed that different ceramide types are significantly associated with final outcome in patients with coronary artery disease [114,115]. The above clinical studies support the further evaluation of modulatory effects of clinical lipid-lowering agents on ceramide to modulate CVD. Researchers found that total left ventricular (LV) glutathione depletion in failing hearts was 54%, and that oral administration of the glutathione precursor N-acetylcysteine (NAC) inhibited neutral sphingomyelinase (N-SMase), Bcl-2 depletion, and caspase-3 activation, normalized LV glutathione, improved LV systolic function, and reduced adverse LV remodeling in rats after MI. These data suggest that recovery from post-infarction heart failure can be achieved by blocking N-SMase activation through NAC supplementation [127]. NAC has already completed multiple phase IV trials regarding its antioxidant effects, one of which is to reverse the changes in thyroid hormones seen in critical illness such as ischemic heart disease. Small-molecule tetrahydroxybutylimidazole (THI) is a food additive approved by the FDA to inhibit S1PL [94]. Irina A Gorshkova et al. found that a lack of SphK1 and low tissue/circulating S1P levels in SphK1 KO mice resulted in poor animal resuscitation after cardiac arrest and impaired survival after resuscitation. Inhibition of S1P lyase by THI in SphK1 KO mice substantially improved animal resuscitation and survival [112]. THI has not been adopted in any clinical trials, but has recently been used as treatment in a mouse model of Huntington’s disease by altering sphingolipid metabolism [128]. These data provide evidence for a critical role of the SphK1 and S1P signaling systems in resuscitation and survival after cardiac arrest, which may lay the foundation for the development of new therapeutic strategies to support resuscitation and long-term survival in cardiac arrest patients.

### 5.2. Bioactive Molecules Regulating Ceramide Metabolism

Researchers have identified several biologically active molecules that have not been used clinically and can affect the cardiovascular disease process by acting on enzymes or synthetic products of the ceramide synthesis pathway.

#### 5.2.1. Molecules Targeting de Novo Synthesis

As an important pathway for ceramide synthesis, the existing drugs targeting the de novo pathway focus on the enzymes SPT and DES. 5′ Adenosine monophosphate-activated protein kinase (AMPK) possesses beneficial properties as an energy sensor for reducing lipotoxicity. Notoginsenoside R1 (NGR1), extracted from the Chinese herb *Panax notoginseng*, upregulates p-AMPK expression and inhibits SPT activity, thereby reducing ceramide expression, promoting fatty acid oxidation in vivo, and substantially improving cardiac function in CHF mice [129]. Investigators have experimentally demonstrated that CIN038 acts as a potent and selective inhibitor of Des1, and attenuates hypertrophy and collagen synthesis in neonatal rat cardiac myocytes by restoring sphingolipid imbalance and reducing the inflammatory response [80]. DEGS1 is the desaturase that catalyzes the final step of the major ceramide biosynthetic pathway. Functional inhibition of DEGS1 activity ameliorates ceramide overexpression. C8-cyclopropenylceramide (C8-CPPC), a selective inhibitor of DEGS1, reduces the expression of pro-adipogenic transcription factors, such as PPARγ and C/EBPβ, as well as adipogenic genes, thereby ameliorating lipid metabolism disorders [81]. The compounds mentioned above, NGR1, CIN038, and C8-CPPC, have been investigated mechanistically at the animal and cellular levels; however, they have not yet entered clinical trials, and need to be explored in depth.

#### 5.2.2. Molecules Targeting the Sphingomyelin Pathway

Currently, the compounds acting on the sphingomyelin pathway are primarily targeted towards SMase and SMS. D-609, an inhibitor of sphingomyelin synthase, substantially reduces cardiac ceramide content after 5 min of ischemia, decreases post-ischemic DNA damage and cardiomyocyte apoptosis, and may reduce infarct size [130,131]. Scyphostatin (SCY) is a specific inhibitor of mammalian neutral magnesium-dependent sphingomyelinase activity. Inhibition of neutral sphingomyelinase (N-SMase) activity by SCY would lead to a decrease in cytokine/chemokine and ceramide levels, along with the inhibition of NF-κB activation and protection of myocardial tissues and cells [132].

#### 5.2.3. Molecules Targeting the Salvage Pathway

The compounds targeting the salvage pathway have been well studied, and found to act on CDase, SPHK, and S1PR. Studies have shown that ANG II stimulates ceramide formation by activating N-SMase, leading to impaired vasoconstriction. Thus, ANG II-induced activation of N-SMase may contribute to the development of vascular dysfunction. N-oleoylethanolamine (OEA), an inhibitor of CDase activity, enhances ANG II- or N-SMase-induced renal vasoconstriction, and leads to ceramide accumulation [133].

In cardiac ischemia/reperfusion injury, Sphk inhibitors (SKIs) resulted in marked changes in the sphingolipid balance, leading to the proportional decrease in the intracellular S1P levels and concomitant increase in ceramide levels, thereby exacerbating reactive-oxygen-species-mediated apoptosis [111]. N,N-Dimethylsphingosine (DMS) is considered an inhibitor [133] of sphingosine kinase (SphK), a key enzyme responsible for the formation of S1P. Studies have confirmed that exogenously administered DMS generates a biphasic response to SphK activation. When DMS was administered at high concentrations (e.g., 10 μM), it inhibited SphK activity and attenuated the myocardial protective effect produced by ischemic preconditioning. In contrast, at low concentrations (1 μM), DMS activated SphK activity via PKCε, producing a cardioprotective effect [134]. PF543, a Sphk1-specific pharmacological inhibitor, was administered as a 14-day infusion of Ang II to C57BL6/J male mice with simultaneous intraperitoneal administration of PF543. Subsequent studies confirmed that PF543 improved endothelial function in the arteries of hypertensive mice by decreasing endothelial-type nitric oxide synthase phosphorylation, while also reducing cardiac hypertrophy and increasing cardiac sphingomyelin (i.e., Sphk1 substrate) levels in vivo. Mechanistically, it was observed that PF543 downregulated Rho-associated helix-coil-containing protein kinase 1, STAT3, protein kinase C (PKC), and ERK1/2 signaling/phosphorylation, effectively regulating cardiac-hypertrophy-related pathways. These results render the pharmacological inhibition of Sphk1 a potential strategy for the control of hypertensive cardiovascular disease [135]. Researchers found that cardiomyocytes with Sphk-1 gene deletion were more susceptible to hypoxia and glucose deprivation stress, and that the monoganglioside GM-1, which activates SphK-1 via PKCε, reduced cardiomyocyte mortality after ischemia/reperfusion injury; the enhanced survival effect produced by GM-1 was abolished when 300 nM of the S1P1 receptor selective antagonist VPC23019 was used [136].

VPC23019 is an aryl-hydrocarbon-containing S1P receptor modulator that specifically targets S1P1 and three GPCRs, rendering them ineffective for preconditioning after ischemia/reperfusion injury in rat cardiomyocytes [137]. S1P2 can promote endothelial cell inflammatory activation, and ONO-5430514 is a specific S1P2 antagonist. Animal experiments using apolipoprotein E-deficient mice have shown that ONO-5430514 attenuates endothelial dysfunction and prevents atherosclerosis, making it a target for the treatment of atherosclerosis [138]. CYM-51736 is an S1P3R-specific agonist; using an in vitro ischemia/reperfusion injury heart model, researchers found that myocardial injury caused by ischemia/reperfusion was ameliorated by the specific activation of S1P3, and concluded that specific drug targeting of the S1P3 receptor could provide therapeutic benefit in ischemic heart disease without the adverse effects of overall activation of other cardiac S1P receptors [139].

## 6. Summary

Numerous studies have elucidated the role and mechanisms of sphingolipids in CVD [9]. Ceramides, as important biologically active metabolites of sphingolipids, are accumulated during disease progression, and exhibit high disease prediction and relevance [140]. Few clinical agents target ceramides, and the underlying mechanisms remain elusive. Although cholesterol is a commonly used clinical biomarker for CVD, it took approximately 50 years for cholesterol-lowering drugs (e.g., statins) to be approved as atherosclerotic CVD drugs [141]. Comparative laboratory and clinical studies using ceramide-targeted drugs for the treatment of cardiac disease still have a long way to go.
